# Effect of ginseng stem leaf extract on the production performance, meat quality, antioxidant status, immune function, and lipid metabolism of broilers

**DOI:** 10.3389/fvets.2024.1463613

**Published:** 2024-09-05

**Authors:** Peng Zhang, Haoyue Zhang, Chuanjie Ma, Qiufeng Lv, Haiyang Yu, Qiang Zhang

**Affiliations:** ^1^College of Life Engineering, Shenyang Institute of Technology, Fushun, China; ^2^College of Animal Science and Veterinary Medicine, Shenyang Agricultural University, Shenyang, China; ^3^Liaoning Zhongqing Xinze Biotechnology Co. Ltd., Huludao, China

**Keywords:** broilers, ginseng stem leaf extract, production performance, meat quality, antioxidant, immunity, blood lipids

## Abstract

**Introduction:**

The present study explores the effect of ginseng stem leaf (GSL) extract on the production performance, meat quality, antioxidant status, immune function, and lipid metabolism of white feathered broilers.

**Methods:**

There were 6 replicates in each group, with 10 broilers in each replicate. In the 42 day trial, 300 AA broilers were randomly divided into five groups: control group (CON), 1.25% GSL extract group (GSL-L), 2.5% GSL group (GSL-M), 5% GSL group (GSL-H), and 45 mg/kg chlortetracycline group (CTC).

**Results:**

The results showed that different doses of GSL extract could improve the body weight, feed to gain ratio (F/G), average daily feed intake (ADFI), average daily gain (ADG), and meat quality of broilers. Compared with the control group, the addition of different doses of GSL improved the antioxidant and immune abilities of broilers to varying degrees, and the effect of GSL extract was significant in the GSL-H group (*p* < 0.05). In addition, medium and high doses of GSL extract significantly reduced the blood triglyceride (TG) and total cholesterol (TC) contents of broilers (*p* < 0.05).

**Discussion:**

Adding GSL extract to the feed has a positive impact on the body weight, meat quality, antioxidant capacity, immunity, and blood lipids of broilers.

## Introduction

*Panax L*. (the ginseng genus) is a group of medicinal plants within the family Araliaceae and all of its species possess broad application prospects. Among them, ginseng (*Panax ginseng* C. A. Mey.) is known as the “King of Herbs” in traditional Chinese medicine by virtue of its high medicinal and nourishing value. Ginsenoside, as one of the major bioactive components in *Panax ginseng*, has attracted extensive research interest. In recent years, chemical, pharmacological, and clinical studies have proven that ginseng stem leaf (GSL) saponins have similar pharmacological effects to ginsenosides. Ginseng stems and leaves contain abundant saponins, polysaccharides, volatile oils, organic acids, proteins, flavonoids, steroids, and other active ingredients (1), which can significantly the enhance immune function. By comparing the effect of different parts of five-year-old ginseng (root/stem/leaf/flower/seed) on the immune system of immunosuppressed mice, some scholars have found that ginseng roots, leaves, and flowers can improve the viability of natural killer (NK) cells, enhance the immune organ index, improve cell mediated immune response, increase the content of CD4+ and the ratio of CD4+/CD8+, and restore the function of macrophages, while ginseng stems and seeds can enhance the thymus index, carbon clearance, splenocyte proliferation, NK cell viability, and IL-4 level of immunosuppressed mice (2). In addition, scholars have designed the compatibility of different compounds with ginsenosides to promote mouse immune response (3, 4), enhance intestinal mucosal immunity (5), and elevate vaccine efficacy (6). Total saponins of GSL can also improve the composition of intestinal flora, increase the abundance of probiotics, and thus enhance the secretion of intestinal IgA response (7). In terms of antiinfection, ginseng can inhibit the formation of bacterial biofilms and induce the dispersion and dissolution of mature biofilms (8). In addition, *in vitro* and *in vivo* studies have revealed the anti-influenza properties of ginseng and its derivatives. Ginsenoside compounds, especially Rb1, interact with hemagglutinin (HA) on the surface of influenza A virus and interfere with the binding of the virus to cell surface receptors, thereby inhibiting the invasion of the virus into host cells (9). In addition to Rb1, scholars have also demonstrated the anti-inflammatory activity of Rg1 (10), the hypoglycemic effect of Rb3 (11), the improving effect of Rb (Rb1, Rb2, Rb3) on the structure and diversity of human intestinal flora (12), as well as the functionality of Rg3 and Rh2 in anti-inflammation, immune regulation, and antioxidation (13), and Rd. is also reported to be a natural neuroprotective agent (14). All these studies indicate that GSL and its effective component saponins contribute to exerting anti-inflammatory response, improving intestinal flora, and regulating immune function. The main bioactive saponins of ginseng exist in the roots, stems, leaves, flowers, and berries, but the stems and leaves of ginseng are not fully utilized. In recent years, due to intensive feeding and environmental biological pollution, the potential application value of GSL in poultry production has been gradually highlighted. Yu et al. (15) found that total saponins from GSL significantly inhibited cyclophosphamide-induced oxidative stress in broilers and induced immune response of Newcastle disease virus (ND) and avian influenza (AI) bivalent inactivated vaccines (16). In addition, Yuan found that after injection of ND virus vaccine into broilers, the absorption rate of GSL saponin (E515-D)-adjuvanted vaccine was faster than that of Marcol 52 and #10 white oil-adjuvanted vaccine (17). Ginseng extract can reduce the negative physiological effects caused by heat stress during broiler breeding (18). Adding 3% red ginseng residue in the diet can significantly decline the mortality and serum cholesterol of chicks and also improve the meat quality of broilers (19). These results indicate that ginseng and its effective components have high application value in broiler feeding.

The residue and drug resistance of antibiotics in meat products have increasingly attracted public attention. As a kind of “safe, efficient, stable, and controllable” feed additive, plant extract has become the first choice of alternative anti-bacterial products in the breeding industry due to its advantages of less drug resistance, small toxic and side effects, low residue and anti-oxidation, which has also been widely promoted in the animal food production and feed industry in developed countries such as Europe and the United States. Numerous data have confirmed the high bactericidal and anti-bacterial properties of plant extracts (20), which can effectively eliminate poultry parasites (21) and other harmful pathogens and even exert excellent killing effect on some drug-resistant strains (22), thereby effectively protecting the intestinal health of animals, enhancing the digestive and immune systems, improving daily weight gain, and achieving antibiotic free and green breeding. Against the background of “total ban on antibiotics,” taking GSL and its extracts as efficient feed additives has great practical significance for expanding and deepening the utilization of traditional Chinese medicine resources and promoting the green development of animal husbandry.

Therefore, the aim of this study was to verify that dietary GSL supplementation can replace feeding antibiotics and improve growth performance, meat quality, antioxidant status, immune function, and lipid metabolism of broilers.

## Materials and methods

### Experimental design and diet

This study was carried out in the animal experiment center of Shenyang Institute of Technology and got approval of the Animal Ethics Committee of College of Life Engineering, Shenyang Institute of Technology (Fushun, China, SITLLBA2024011). GSL extract (80.6% saponin, 0.02% crude fiber, 1.1% ash content, and 2.2% water) was purchased from SKY ENERGY Biotechnology Co., Ltd. A total of 300 1-day-old AA broilers (average weight 0.04 kg) were randomly divided into five treatment groups, with six replicates in each treatment group and 10 broilers (5 males and 5 females) in each replicate. The poultry house was fully rinsed and fumigated before the experiment. The experimental broilers were caged in upper, middle, and lower layers (1.75 long × 1.55 wide × 0.5 high, m). The temperature of the poultry house was raised to 32 ~ 35°C 24 h in advance, and then, the temperature was reduced by 2 ~ 3°C every week until it was maintained at 21 ~ 24°C, with continuous light and natural ventilation. The animals had free access to food and drinking water throughout the experimental period and were immunized according to the standardized procedures. The dietary treatments were divided into CON (i.e., basal diet), GSL-L (1.25% of GSL extract in the basal diet), GSL-M (2.5% of GSL extract in the basal diet), GSL-H (5% of GSL extract in the basal diet), and CTC (basic diet supplemented with 45 mg/kg chlortetracycline). The experiment lasted 42 days in total, with 1–21 days as the early stage and 22–42 days as the later stage. The relative humidity of the chicken house was controlled at 50 ~ 70% during the entire experiment. The basal diet for the experiment was corn-soybean meal. The formula was based on NRC (1994) and Chicken Feeding Standard (NY/T33-2004). Table 1 shows the composition and nutritional level of basal diet.

**Table 1:** Composition and nutritional level of basal diet (air-dried).

Item	1-21d	22-42d
Diet composition %		
Corn	63.0	64.00
Wheat bran	1.68	1.95
Soybean meal	27.00	28.40
Fish meal	3.00	0
Soybean oil	1.40	1.60
Limestone	1.42	1.45
Dicalcium phosphate	0.60	0.7
Salt	0.30	0.3
Choline	0.10	0.1
Premix^1^	1.50	1.50
Total	100	100
Nutrient levels^2^		
ME (MJ/kg)	12.99	12.36
CP (%)	18.45	17.11
Lys (%)	0.77	0.88
Met (%)	0.34	0.33
Ca (%)	0.80	0.80
TP (%)	0.53	0.41

^1Premix provided per kilogram of diet: VA 12000 IU, VD3 2,500 IU, VE 21.0 mg, VK3 3.0 mg, VB1 5.0 mg, VB2 9.0 mg, VB6 8.0 mg, VB12 2.03 mg, pantothenic acid 20.0 mg, niacin 50.0 mg, biotin 0.1 mg, folic acid 1.5 mg, Fe 94 mg, Cu 23 mg, I 0.7 mg, Zn 92 mg, Mn 101.4 mg, Se 0.04 mg. 2Calculated value.^

### Sample collection

The blood of broilers was collected in the morning of the 42nd day, and one broiler was randomly selected from each replicate. Approximately 10 mL of blood was extracted from the vein under the chicken wing using a 5 mL syringe and centrifuged at 4000 × g for 10 min to isolate the serum, and then stored at-20°C for subsequent analysis. After blood collection, the broilers were sacrificed by cervical dislocation. The whole thigh (biceps femoris) and chest (pectoralis major) musculature were removed from the same broiler. The meat quality traits of thigh and chest stored at 4°C were analyzed. The abdominal cavity of broilers was opened after death from bloodletting, and the liver tissues were taken and stored in liquid nitrogen in a cryopreservation tube for future use.

### Growth performance and organ index

Broilers in each replicate were weighed on days 21 and 42, and the feed consumption of each replicate was recorded on days 1–21 and 22–42. Average daily gain (ADG), average daily feed intake (ADFI), and feed to gain ratio (F/G) were calculated for each replicate. On the 42nd day, the weight of one broiler from each replicate was close to the average weight, followed by cervical dislocation. Immune organs (liver, spleen, thymus, and bursa of Fabricius) were collected to calculate the percentage of organs.

### Meat quality

The samples of pectoral and thigh muscles on both sides of each broiler were collected. Within 12 h after death, the pH values of biceps femoris and pectoralis major muscles were measured using a penetrating electrode (Mettler Toledo, Changzhou, China) connected to a portable pH meter (FG2, Shanghai, China). The pH probe was calibrated with pH = 4 and pH = 7 standard buffer solutions before testing. Meat color (L* = relative lightness, a* = relative redness, and b* = relative yellowness) was measured with a handheld color reader (CR10, Konica Minolta INC, Osaka, Japan).

The reading from the inner surface of the sample represented the entire surface of the muscle, with white tiles (L*92.30, a*0.32, and b*0.33) as standards. Approximately 20 g of samples from thigh and chest muscles were used to determine the drip loss. Muscle samples were weighed and fixed in plastic bags filled with air to avoid evaporation, suspended in a 4°C cooler for 24 h, and then weighed again. The drip loss was calculated according to the weight loss and expressed as a percentage. After pH and drip loss measurements, the pectoral muscle samples were loaded into plastic bags and placed at room temperature before cooking. Then, the muscle samples were heated to an internal temperature of 70°C in a boiling water bath. The cooked muscle was cooled to room temperature and cut into pieces of 3 × 1 × 1 cm3, parallel to the direction of the muscle fibers. The Warner-Bratzler shear force was assessed using a texture measurement system (Food Technology Corporation, Stirling, VA, USA).

### Antioxidant capacity

About 0.3 g of liver samples stored in liquid nitrogen were put into a centrifuge tube, added with 0.9% normal saline nine times the weight of the sample, and homogenized at 4000 rpm/min for 10 min under the condition of ice water bath, and the supernatant was sucked with a pipette gun. The total antioxidant capacity (T-AOC) (A015-2-1), superoxide dismutase (T-SOD) (A001-3-2), glutathione peroxidase (GSH-PX) (A005-1-2), catalase (CAT) (A007-1-1), and malondialdehyde (MDA) (A003-1-2) were detected by corresponding kits. The absorbance at 425 nm and 520 nm was determined by a microplate reader (Bio-Tek ELx808). The above kits were purchased from Nanjing Jiancheng Bioengineering Co., Ltd. (Nanjing, China).

### Detection of serum immune indexes

The sample serum was used for the detection of IgA (m1002792V), IgG (m1042771V), and IgM (m1027781V) according to the kit instructions. The kits were purchased from Shanghai Meilian Biotechnology Co., Ltd. (Shanghai, China), and the absorbance at 450 nm was detected with a microplate reader.

### Analysis of biochemical and lipid indicators

The concentrations of total cholesterol (TC) (A111-1-1), triglycerides (TG) (A110-1-1), high-density lipoprotein cholesterol (HDL) (A112-1-1), and low-density lipoprotein cholesterol (LDL) (A113-1-1) in serum samples were detected using commercial kits (Nanjing Jiancheng Bioengineering Co., Ltd., Nanjing, China). The absorbance at 500 nm and 600 nm was detected with a microplate reader.

### Statistics analysis

The experimental data were collated with Excel 2019, and SPSS 26.0 statistical software was used for one-way analysis of variance (ANOVA). Duncan’s multiple comparisons were performed on the data with significant differences. *p* < 0.05 and *p* < 0.01 are regarded as significant differences and extremely significant differences, respectively, while 0.05 < *p* < 0.1 is indicative of a trend. Data are expressed as mean ± standard deviation.

## Results

### Effect of GSL extract on body weight of white feathered broilers

The effect of GSL extract on the body weight of white feathered broilers is shown in Table 2. At 21 and 42 days, there was no significant difference in the body weight of broilers in each group (*p* > 0.05), but with the increase of the dose of GSL extract, the body weight also showed a linear growth trend. In addition, the F/G of the high-dose group was significantly higher than that of the control group and CTC group (*p* < 0.05). Although there was no difference in ADFI and ADG among the groups (*p* > 0.05), the ADFI and ADG of GSL extract groups had an upward trend with the increase of dose.

**Table 2:** Effect of GSL extract on body weight of white feathered broilers.

Item	Group				
	CON	GSLL	GSLM	GSLH	CTC
21d (kg)	0.83 ± 0.07	0.86 ± 0.09	0.87 ± 0.06	0.88 ± 0.04	0.82 ± 0.09
42d (kg)	3.32 ± 0.14	3.31 ± 0.15	3.33 ± 0.17	3.34 ± 0.11	3.31 ± 0.21
ADFI (g)	85.3 ± 3.2	84.3 ± 3.7	85.2 ± 5.0	85.4 ± 4.1	85.2 ± 3.9
ADG (g)	35.7 ± 2.2	36.3 ± 1.4	36.5 ± 2.0	36.9 ± 2.1	35.8 ± 1.1
F/G	2.63 ± 0.11^b^	2.69 ± 0.09^ab^	2.74 ± 0.12^ab^	2.77 ± 0.07^a^	2.66 ± 0.07^b^

In the same row of data, different small letters indicate significant differences (*p* < 0.05); the same small letters or no letters indicate no significant differences (*p* > 0.05). CON, basal diet; GSL-L, CON + 1.25% GSL extract; GSL-M, CON + 2.5% GSL extract; GSL-H, CON + 5% GSL extract; CTC, CON + 45 mg/kg chlortetracycline.

### Effect of GSL extract on meat quality of white feather broilers

The effect of GSL extract on the meat quality of white feather broilers is shown in Table 3. Compared with the control group, the medium-dose and high-dose groups showed significantly reduced chicken shearing force (*p* < 0.05), and the pH value of chicken in the high-dose group was significantly decreased (*p* < 0 0.05), but there was no significant difference among other groups. The drip loss of the high-dose group was significantly lower than that of other groups (*p* < 0.05). The cooking loss of the high-dose group and CTC group was significantly lower than that of the control group and low-dose group (*p* < 0.05). Compared with the control group, the a* of the medium-dose group and high-dose group were significantly decreased (*p* < 0.05), but there was no significant difference among other groups (*p* > 0.05). L* and b* showed a linear correlation with the concentration of GSL extract, and there was no significant difference in L* and b* among the groups (*p* > 0.05).

**Table 3:** Effect of GSL extract on meat quality of white feather broilers.

Item	Group				
	CON	GSLL	GSLM	GSLH	CTC
shear force *N*	9.45 ± 0.29^a^	8.53 ± 0.23^ab^	6.76 ± 0.25^b^	6.11 ± 0.25^b^	8.55 ± 0.38^ab^
pH(24 h)	5.64 ± 0.23^a^	5.62 ± 0.30^ab^	5.56 ± 0.31^ab^	5.52 ± 0.22^b^	5.74 ± 0.25^ab^
drip loss %	5.30 ± 0.59^a^	5.06 ± 0.50^a^	4.84 ± 0.27^a^	4.40 ± 0.44^b^	4.96 ± 0.54^a^
pressure loss %	21.73 ± 1.37^a^	20.44 ± 1.67^a^	17.54 ± 1.55^ab^	14.46 ± 1.07^b^	15.03 ± 1.06^b^
L*	57.44 ± 2.60	57.81 ± 2.12	56.91 ± 2.25	57.75 ± 1.75	57.05 ± 3.63
a*	4.36 ± 1.06^a^	3.98 ± 1.77^ab^	3.10 ± 1.08^b^	3.00 ± 0.74^b^	3.43 ± 0.82^ab^
b*	15.33 ± 3.49	13.65 ± 2.24	14.57 ± 1.50	14.30 ± 2.40	14.07 ± 2.98

In the same row of data, different small letters indicate significant differences (*p* < 0.05); the same small letters or no letters indicate no significant differences (*p* > 0.05). CON, basal diet; GSL-L, CON + 1.25% GSL extract; GSL-M, CON + 2.5% GSL extract; GSL-H, CON + 5% GSL extract; CTC, CON + 45 mg/kg chlortetracycline.

### Effects of GSL extract on antioxidant indexes of white feather broilers

The effect of GSL extract on antioxidant indexes of white feather broilers is shown in Table 4. Compared with the control group, the CAT activity in the high-dose group was significantly increased (*p* < 0.05), and the CAT activity increased linearly with the increase of the dose of GSL extract. Compared with the control group, the activities of T-SOD and T-AOC were significantly increased in the high-dose group (*p* < 0.05). The activities of T-SOD and T-AOC in other experimental groups were enhanced with the increasing dose of GSL extract, and the activity of T-AOC in the CTC group was significantly lower than that in the high-dose group (*p* < 0.05). Compared with the control group, the content of MDA in the medium-dose group and high-dose group was significantly decreased (*p* < 0.05), and the differences in the content of MDA among the other groups were not significant, but the content of MDA showed a decreasing trend (*p* > 0.05). Although there was no significant difference in the content of GSH-PX among the groups (*p* > 0.05), it also increased with the increasing dose of GSL extract.

**Table 4:** Effects of GSL extract on antioxidant indexes of white feather broilers.

Item	Group				
	CON	GSLL	GSLM	GSLH	CTC
GSH-PX (U/mg)	33.51 ± 3.10	34.78 ± 3.68	35.18 ± 3.66	37.38 ± 3.63	37.75 ± 3.87
CAT (U/mg)	4.86 ± 0.58^b^	5.05 ± 0.77^ab^	5.28 ± 0.60^ab^	5.31 ± 0.67^a^	5.16 ± 0.62^ab^
T-SOD (U/mg)	198.40 ± 8.46^b^	226.84 ± 8.78^ab^	241.72 ± 9.06^ab^	285.87 ± 8.70^a^	217.70 ± 9.13^ab^
T-AOC (mmol/g)	0.11 ± 0.02^b^	0.20 ± 0.02^ab^	0.21 ± 0.02^ab^	0.22 ± 0.02^a^	0.12 ± 0.03^b^
MDA (nmol/mg)	2.35 ± 0.32^a^	1.97 ± 0.36^ab^	1.67 ± 0.34^b^	1.57 ± 0.35^b^	1.76 ± 0.33^ab^

Data are represented as the mean ± SD (*n* = 6). ^a,b^Values within a row with different superscript letters indicate significant differences (*p* < 0.05) among groups, and the same superscript letters or no superscript letters indicate non-significant differences (*p* > 0.05). T-AOC: total antioxidant capacity; CAT: catalase; MDA: malondialdehyde; GSH-Px: glutathione peroxidase; T-SOD: superoxide dismutase.

### Effect of GSL extract on immune function of white feather broiler

Table 5 shows the effect of GSL extract on immune organ indexes of white feather broilers. Compared with the control group, the bursa of Fabricius index in the high-dose group and CTC group was increased significantly (*p* < 0.05). Compared with the high-dose group, the thymus index of the medium-dose group and low-dose group was decreased significantly (*p* < 0.05), and there was no significant difference among other groups (*p* > 0.05). There was no significant difference in spleen index among the groups (*p* > 0.05).

**Table 5:** Effect of GSL extract on immune organ index of white feathered broilers.

Item	Group				
	CON	GSLL	GSLM	GSLH	CTC
Thymus organ index (g/kg)	2.20 ± 0.07^ab^	1.88 ± 0.09^bc^	1.95 ± 0.07^bc^	2.42 ± 0.11^a^	2.19 ± 0.08^ab^
Bursa organ index (g/kg)	1.16 ± 0.04^c^	1.19 ± 0.06^bc^	1.43 ± 0.06^abc^	1.58 ± 0.05^a^	1.52 ± 0.05^ab^
Spleen organ index (g/kg)	0.10 ± 0.02	0.11 ± 0.03	0.11 ± 0.03	0.10 ± 0.02	0.16 ± 0.05

Data are represented as the mean ± SD (*n* = 6). ^a,b^Values within a row with different superscript letters indicate significant differences (*p* < 0.05) among groups, and the same superscript letters or no superscript letters indicate non-significant differences (*p* > 0.05).

The effect of GSL extract on immunoglobulin indexes of white feathered broilers is shown in Table 6. Compared with the control group, the IgA content and IgG content in the medium-dose group and high-dose group were significantly increased (*p* < 0.05). In addition, compared with the CTC group, the IgG content in the high-dose group and the medium-dose group was increased significantly (*p* < 0.05). There was no significant difference in the content of IgM among the groups (*p* > 0.05), but with the increase of the dose of GSL extract, the content of IgM also showed an upward trend.

**Table 6:** Effect of GSL extract on immunoglobulin of white feather broilers.

Item	Group				
	CON	GSLL	GSLM	GSLH	CTC
IgA (μg/mL)	15.99 ± 2.17^b^	25.67 ± 2.78^ab^	34.42 ± 2.11^a^	36.42 ± 2.21^a^	25.75 ± 2.40^ab^
IgM (μg/mL)	141.96 ± 9.18	148.21 ± 8.56	161.66 ± 8.64	206.06 ± 8.79	191.56 ± 8.43
IgG (μg/mL)	361.28 ± 18.39^b^	457.53 ± 18.04^b^	497.69 ± 16.81^a^	534.41 ± 19.90^a^	406.28 ± 19.46^b^

Data are represented as the mean ± SD (*n* = 6). ^a,b^Values within a row with different superscript letters indicate significant differences (*p* < 0.05) among groups, and the same superscript letters or no superscript letters indicate non-significant differences (*p* > 0.05). IgA, Immunoglobulin A; IgG, Immunoglobulin G; IgM, Immunoglobulin M.

### Effect of GSL extract on blood lipid metabolism of white feather broilers

Table 7 shows the effect of GSL extract on blood lipid indexes of white feather broilers. Compared with the control group, the contents of TC and TG in the medium-dose group, high-dose group, and CTC group were significantly decreased (*p* < 0.01). The HDL-C and LDL-C in the CTC group were significantly lower than those in the control group (*p* < 0.05), and there was no significant difference among other groups.

**Table 7:** Effect of GSL extract on blood lipid indexes of white feathered broilers.

Item	Group				
	CON	GSLL	GSLM	GSLH	CTC
TC (mmol/L)	0.29 ± 0.04^A^	0.25 ± 0.10^AB^	0.17 ± 0.07^BC^	0.18 ± 0.07^BC^	0.12 ± 0.06^C^
HDL-C (mmol/L)	4.65 ± 0.11^a^	4.27 ± 0.15^ab^	4.24 ± 0.13^ab^	3.91 ± 0.14^ab^	3.51 ± 0.11^b^
LDL-C (mmol/L)	1.26 ± 0.07^a^	0.93 ± 0.07^ab^	1.11 ± 0.06^ab^	1.03 ± 0.07^ab^	0.82 ± 0.07^b^
TG (mmol/L)	3.73 ± 0.04^A^	3.51 ± 0.03^A^	2.66 ± 0.02^B^	2.70 ± 0.03^B^	2.53 ± 0.01^B^

Data are represented as the mean ± SD (*n* = 6). ^a,b^Values within a row with different superscript letters indicate significant differences (*p* < 0.05) among groups, and the same superscript letters or no superscript letters indicate non-significant differences (*p* > 0.05). ^A,B^Values within a row with different superscript letters indicate significant differences (*p* < 0.01) among groups, and the same superscript letters or no superscript letters indicate non-significant differences (*p* > 0.01). HDL: high-density lipoprotein; LDL: low-density lipoprotein; TG: triacylglycerol; TC: total cholestero.

## Discussion

The synergistic treatment of feed and plant extract can reduce the anti-nutritional effect of anti-nutritional factors in feed, thus promoting the digestibility and utilization of energy and nutrients in broilers, elevating feed remuneration, and improving animal production performance (23, 24). GSL extract is rich in saponins and flavonoids, which can improve the production performance, immune function, and meat quality of quails (25). However, the effect of GSL extract on the production performance of white feathered broilers is not yet clear. Our results showed that the addition of GSL extract to the diet had no significant effect on the body weight of white feathered broilers, but the body weight increased with the increasing dose of GSL extract. After adding GSL extract, both ADG and F/G showed an upward trend, and the F/G of the group added with 5% GSL extract was significantly higher than that of the control group (*p* < 0.05), which was similar to the research results of Liu et al. (26) that adding ginseng extract increased the ADG and F/G of broilers. Saponins in American ginseng and ginseng can inhibit pancreatic lipase activity and reduce body weight, adipose tissue weight, and blood lipids of high-fat diet mice, thereby controlling obesity caused by high-fat diet (27, 28). The blood lipid indexes can comprehensively reflect the ab-sorption and metabolism of starch, lipids, and other nutrients in the feed of broilers (29). In this study, by detecting the cholesterol metabolism indexes in the blood of broilers, it was found that the TC and TG in the blood of broilers fed GSL extract were significantly reduced (*p* < 0.05). Kim M H (30) et al. found that adding 1% red ginseng to the diet could reduce TC and LDL-C, but not HDL-C in hyperlipidemic mice, which was in consistence with our experimental results. Collectively, these findings suggest that GSL extract can significantly improve the growth performance of white feathered broilers, especially in lowering lipid and increasing F/G.

pH value, drip loss, and shear force are the main physical indicators to evaluate meat quality (31). It is reported that the saponin component in ginseng extract pro-motes angiogenesis, which can rapidly regenerate the blood vessels of limbs and relieve vascular injury or local hypoxia (32). The development of blood vessels improves the efficiency of nutrient transport, accelerates the metabolism of limbs, and promotes the normal development of pectoral and thigh muscles (33). Therefore, we speculate that GSL extract can prevent the lignification of chicken breast meat and the growth restriction of acral tissues caused by long-term high-density breeding conditions. Our results demonstrated that GSL extract reduced the pH value, drip loss, and muscle shear force of chicken, thereby effectively improving meat quality. Similar results were also found by Morsy et al. (34) that adding 300 mg/kg ginseng extract to the diet improved the shear force of chicken during the growth period and reduced the pH value and drip loss.

Intensive feeding can lead to the production of excessive reactive oxygen free radicals in broilers. Therefore, it is of practical significance to develop feed additives that can reduce lipid peroxide, eliminate oxygen free radicals, and enhance disease resistance of broilers (35). SOD, CAT, and GSH-PX are essential for reducing excessive reactive oxygen radicals (36). GSL extract exerts its antioxidant effect mainly by regulating the production of SOD and GSH-PX, thereby indirectly affecting the antioxidant capacity of the body (37). In our experiment, adding 5% GSL extract to the basal diet could significantly increase the activities of T-SOD, T-AOC, and CAT in the liver of white feathered broilers and reduce the content of MDA in the liver (*p* < 0.05). Jiao et al. (38) also reported that ginseng extract enhanced SOD and T-AOC activities in the se-rum and liver of D-galactose-induced aging mice, while reducing MDA content. *In vitro* and *in vivo* tests have revealed that plant extracts achieve antioxidant effects by reducing the generation of oxygen free radicals and enhancing the activities of antioxidant enzymes such as SOD, GSH-PX, and CAT (39–41). The above results confirm that adding GSL extract to the diet can improve the serum antioxidant capacity of white feathered broilers and reduce oxidative stress damage.

The development of immune organs underlies the realization of immune function, and the immune organ index is an important indicator to measure the immune status of poultry (42). Spleen, thymus, and bursa of Fabricius are important sites for the formation and differentiation of T and B lymphocytes. Therefore, the organ indexes of spleen, thymus, and bursa of Fabricius are commonly used to evaluate the immune status of poultry. The higher the index, the stronger the immune system function (43). Song et al. (44) found that adding 300 mg/kg ginsenoside Rg1 could significantly in-crease the organ indexes of bursa of Fabricius, spleen, and thymus and enhance the immune function of broilers. Our results also showed that adding an appropriate dose of GSL extract to the diet of white feather broilers promoted the development of bursa of Fabricius and improved the immunity, which may be due to that the saponins contained in GSL extract can promote the proliferation of lymphocytes in bursa of Fabricius. Serum IgA, IgG, and IgM, as the most important signs reflecting the humoral immune function, are produced by the proliferation and differentiation of B cells into plasma cells after receiving antigen stimulation. The higher the titer of serum antibody, the stronger the anti-infection and disease resistance ability (45). Our experimental results showed that the addition of GSL extract to the diet improved the bursa of Fabricius index and increased the serum IgG content of white feather broilers. Similar results were revealed by Ma et al. [45] that GSL saponins combined with selenium effectively increased the content of serum IgA after broilers were vaccinated with Newcastle disease vaccine, indicating the potential of GSL extract strengthening immunity of broilers.

Incorporating varying doses of ginseng stem and leaf extracts into the diet can enhance the production performance and meat quality of broilers, while also improving their antioxidant status, cholesterol metabolism, and immune function. These changes indicate the potential application value of ginseng stem and leaf extracts in broiler production. However, to facilitate their widespread use in animal husbandry, further research is required to determine the optimal dosage, action targets, and mechanisms of action of the extracts. Such studies will not only deepen our understanding of how ginseng stem and leaf extracts influence host health but also provide a theoretical basis for the development of novel green feed additives.

## Data Availability

The datasets presented in this study can be found in online repositories. The names of the repository/repositories and accession number(s) can be found in the article/supplementary material.

## References

[1] Hou M Wang R Zhao S Wang Z. Ginsenosides in Panax genus and their biosynthesis. Acta Pharm Sin B. (2021) 11:1813–34. doi: 10.1016/j.apsb.2020.12.017, PMID: 34386322 PMC8343117

[2] Chen LX Qi YL Qi Z Gao K Gong RZ Shao ZJ. Comparative study on the effects of different parts of *Panax ginseng* on the immune activity of cyclophosphamide-induced immunosuppressed mice. Molecules. (2019) 24:1096. doi: 10.3390/molecules24061096, PMID: 30897728 PMC6470474

[3] Wang Y Yuan L Cui X Xu W Fang S Li Z. Ginseng stem-leaf saponins in combination with selenium promote the immune response in neonatal mice with maternal antibody. Vaccine. (2020) 8:755. doi: 10.3390/vaccines8040755 PMC7768402 33322647

[4] Sun F Zhou J Zhang Y Liu Q Wang Q Liu X. A compound ginseng stem leaf saponins and aluminium adjuvant enhances the potency of inactivated *Aeromonas salmonicida* vaccine in turbot. Fish Shellfish Immunol. (2022) 128:60–6. doi: 10.1016/j.fsi.2022.07.027, PMID: 35843529

[5] Yuan L Wang Y Ma X Cui X Lu M Guan R. Sunflower seed oil combined with ginseng stem-leaf saponins as an adjuvant to enhance the immune response elicited by Newcastle disease vaccine in chickens. Vaccine. (2020) 38:5343–54. doi: 10.1016/j.vaccine.2020.05.063, PMID: 32571723

[6] Su F Li J Xue Y Yu B Ye S Xu L. Early Oral Administration of Ginseng Stem-Leaf Saponins Enhances the Peyer’s patch-dependent maternal IgA antibody response to a PEDV inactivated vaccine in mice, with gut microbiota involvement. Vaccine. (2023) 11:830. doi: 10.3390/vaccines11040830, PMID: 37112742 PMC10143706

[7] Iqbal H Rhee DK. Ginseng alleviates microbial infections of the respiratory tract: a review. J Ginseng Res. (2020) 44:194–204. doi: 10.1016/j.jgr.2019.12.001 32148400 PMC7031735

[8] Dong W Farooqui A Leon AJ Kelvin DJ. Inhibition of influenza a virus infection by ginsenosides. PLoS One. (2017) 12:e0171936. doi: 10.1371/journal.pone.0188687, PMID: 28187149 PMC5302443

[9] Wang ZL Chen LB Qiu Z Chen XB Liu Y Li J. Ginsenoside Rg1 ameliorates testicular senescence changes in D-gal-induced aging mice via anti-inflammatory and antioxidative mechanisms. Mol Med Rep. (2018) 17:6269–76. doi: 10.3892/mmr.2018.8659, PMID: 29512726 PMC5928602

[10] Meng F Su X Li W Zheng Y. Ginsenoside Rb3 strengthens the hypoglycemic effect through AMPK for inhibition of hepatic gluconeogenesis. Exp Ther Med. (2017) 13:2551–7. doi: 10.3892/etm.2017.4280, PMID: 28565878 PMC5443165

[11] Zheng F Zhang MY Wu YX Wang YZ Li FT Han MX. Biotransformation of Ginsenosides (Rb1, Rb2, Rb3, Rc) in human intestinal bacteria and its effect on intestinal flora. Chem Biodivers. (2021) 18:e2100296. doi: 10.1002/cbdv.202100296, PMID: 34665516

[12] Xu W Lyu W Duan C Ma F Li X Li D. Preparation and bioactivity of the rare ginsenosides Rg3 and Rh2: An updated review. Fitoterapia. (2023) 167:105514. doi: 10.1016/j.fitote.2023.105514, PMID: 37084851

[13] Chen YY Liu QP An P Jia M Luan X Tang JY. Ginsenoside Rd: a promising natural neuroprotective agent. Phytomedicine. (2022) 95:153883. doi: 10.1016/j.phymed.2021.153883, PMID: 34952508

[14] Yu J Chen Y Zhai L Zhang L Xu Y Wang S. Antioxidative effect of ginseng stem-leaf saponins on oxidative stress induced by cyclophosphamide in chickens. Poult Sci. (2015) 94:927–33. doi: 10.3382/ps/pev055, PMID: 25713395

[15] Yu J Shi FS Hu S. Improved immune responses to a bivalent vaccine of Newcastle disease and avian influenza in chickens by ginseng stem-leaf saponins. Vet Immunol Immunopathol. (2015) 167:147–55. doi: 10.1016/j.vetimm.2015.07.017, PMID: 26277227

[16] Yuan L Wang Y Li Z Ma X Cui X Chi X. Sunflower seed oil containing ginseng stem–leaf saponins (E515-D) is a safe adjuvant for Newcastle disease vaccine. Poult Sci. (2020) 99:4795–803. doi: 10.1016/j.psj.2020.06.063, PMID: 32988514 PMC7598328

[17] Sandner G Mueller AS Zhou X Stadlbauer V Schwarzinger B Schwarzinger C. Ginseng extract ameliorates the negative physiological effects of heat stress by supporting heat shock response and improving intestinal barrier integrity: evidence from studies with heat-stressed Caco-2 cells, C elegans and growing broilers. Molecules. (2020) 25:835. doi: 10.3390/molecules25040835 32075045 PMC7070719

[18] Kim YJ Lee GD Choi IH. Effects of dietary supplementation of red ginseng marc and α-tocopherol on the growth performance and meat quality of broiler chicken. J Sci Food Agric. (2014) 94:1816–21. doi: 10.1002/jsfa.6497, PMID: 24281747

[19] Silva DM Costa PAD Ribon AO Purgato GA Gaspar DM Diaz MA. Plant extracts display synergism with different classes of antibiotics. An Acad Bras Cienc. (2019) 91:e20180117. doi: 10.1590/0001-3765201920180117, PMID: 31090789

[20] Nahed A Abd El-Hack ME Albaqami NM Khafaga AF Taha AE Swelum AA. Phytochemical control of poultry coccidiosis: a review. Poult Sci. (2022) 101:101542. doi: 10.1016/j.psj.2021.101542 34871985 PMC8649401

[21] Yuan W Yuk HG. Antimicrobial efficacy of Syzygium antisepticum plant extract against Staphylococcus aureus and methicillin-resistant S. Aureus and its application potential with cooked chicken. Food Microbiol. (2018) 72:176–84. doi: 10.1016/j.fm.2017.12.002, PMID: 29407395

[22] Zhu N Wang J Yu L Zhang Q Chen K Liu B. Modulation of growth performance and intestinal microbiota in chickens fed plant extracts or virginiamycin. Front Microbiol. (2019) 10:1333. doi: 10.3389/fmicb.2019.01333 31275268 PMC6591263

[23] Ahmadipour B Hassanpour H Khajali F. Evaluation of hepatic lipogenesis and antioxidant status of broiler chickens fed mountain celery. BMC Vet Res. (2018) 14:1–7. doi: 10.1186/s12917-018-1561-6 30103743 PMC6088407

[24] Nan GE Kaiyan XIAO Jiangmu WANG Chi LIU Xinyue LIU Qing MA. Effects of aqueous extract of Panax quinquefolium stems and leaves in diet on growth and meat quality of quails. Fujian J Agric Sci. (2023) 38:1395–404. doi: 10.19303/j.issn.1008-0384.2023.12.002

[25] Liu J Wang H Luo J Chen T Xi Q Sun J. Synergism of fermented feed and ginseng polysaccharide on growth performance, intestinal development, and immunity of Xuefeng black-bone chickens. BMC Vet Res. (2024) 20:13. doi: 10.1186/s12917-023-03859-y 38184589 PMC10770880

[26] Sharmila J Aravinthan A Shin DG Seo JH Kim B Kim NS. GBCK25, fermented ginseng, attenuates cardiac dysfunction in high fat diet-induced obese mice. J Ginseng Res. (2018) 42:356–60. doi: 10.1016/j.jgr.2017.05.001, PMID: 29989028 PMC6035385

[27] Koh EJ Kim KJ Choi J Jeon HJ Seo MJ Lee BY. Ginsenoside Rg1 suppresses early stage of adipocyte development via activation of C/EBP homologous protein-10 in 3T3-L1 and attenuates fat accumulation in high fat diet-induced obese zebrafish. J Ginseng Res. (2017) 41:23–30. doi: 10.1016/j.jgr.2015.12.005, PMID: 28123318 PMC5223064

[28] Yener Y Yalcin S Colpan I. Effects of dietary supplementation of red ginseng root powder on performance, immune system, caecal microbial population and some blood parameters in broilers. Ankara Üniversitesi Veteriner Fakültesi Dergisi. (2021) 68:137–45. doi: 10.33988/auvfd.716897

[29] Kim MH Lee EJ Cheon JM Nam KJ Oh TH Kim KS. Antioxidant and hepatoprotective effects of fermented red ginseng against high fat diet-induced hyperlipidemia in rats. Lab Anim Res. (2016) 32:217–23. doi: 10.5625/lar.2016.32.4.217, PMID: 28053615 PMC5206228

[30] Mueller S Kreuzer M Siegrist M Mannale K Messikommer RE Gangnat IDM. Carcass and meat quality of dual-purpose chickens (Lohmann dual, Belgian Malines, Schweizerhuhn) in comparison to broiler and layer chicken types. Poult Sci. (2018) 97:3325–36. doi: 10.3382/ps/pey172, PMID: 29788213

[31] Leung KW Wong AST. Pharmacology of ginsenosides: a literature review. Chin Med. (2010) 5:1–7. doi: 10.1186/1749-8546-5-20 20537195 PMC2893180

[32] Jang HD Kim HJ Min BJ Cho JH Chen YG Yoo JS. Effects of fermented wild-ginseng culture by-products on growth performance, blood characteristics, meat quality and ginsenoside concentration of meat in finishing pigs. J Anim Sci Technol. (2007) 49:329–40. doi: 10.5187/JAST.2007.49.3.329

[33] Morsy MM Farag EL. Effect of dietary ginseng and ginsenosides supplementation on productive, physiological immunological parameters and meat quality of gimmizah cockerels 1. During rearing period. Egyptian Poultry Sci J. (2018) 38:679–98.

[34] Gulcin İ. Antioxidants and antioxidant methods: An updated overview. Arch Toxicol. (2020) 94:651–715. doi: 10.1007/s00204-020-02689-3, PMID: 32180036

[35] Santos-Sánchez NF Salas-Coronado R Villanueva-Cañongo C Hernández-Carlos B. Antioxidant compounds and their antioxidant mechanism. Antioxidants. (2019) 10:1–29. doi: 10.5772/intechopen.85270

[36] Wang H Peng D Xie J. Ginseng leaf-stem: bioactive constituents and pharmacological functions. Chin Med. (2009) 4:1–8. doi: 10.1186/1749-8546-4-20 19849852 PMC2770043

[37] Lili J Wang M Liu Z Zhang X Liu S. Antioxidant activities of the oligosaccharides from the roots. flowers and leaves of *Panax ginseng* CA Meyer. Carbohydr Polym. (2014) 106:293–8. doi: 10.1016/j.carbpol.2014.02.035 24721081

[38] Kochan E Szymańska G Wielanek M Wiktorowska-Owczarek A Jóźwiak-Bębenista M Grzegorczyk-Karolak I. The content of triterpene saponins and phenolic compounds in American ginseng hairy root extracts and their antioxidant and cytotoxic properties. Plant Cell Tissue Organ Cult. (2019) 138:353–62. doi: 10.1007/s11240-019-01633-3

[39] Zhao G Niu Y Wang H Qin S Zhang R Wu Y. Effects of three different plant-derived polysaccharides on growth performance, immunity, antioxidant function, and cecal microbiota of broilers. J Sci Food Agric. (2024) 104:1020–9. doi: 10.1002/jsfa.12988, PMID: 37718500

[40] Kim HM Song Y Hyun GH Long NP Park JH Hsieh YS. Characterization and antioxidant activity determination of neutral and acidic polysaccharides from *Panax ginseng* CA Meyer. Molecules. (2020) 25:791. doi: 10.3390/molecules25040791, PMID: 32059482 PMC7070964

[41] Wu QJ Zheng XC Wang T Zhang TY. Effects of dietary supplementation with oridonin on the growth performance, relative organ weight, lymphocyte proliferation, and cytokine concentration in broiler chickens. BMC Vet Res. (2018) 14:1–6. doi: 10.1186/s12917-018-1359-6 29386029 PMC5793358

[42] Kenarova B Neychev H Hadjiivanova C Petkov VD. Immunomodulating activity of ginsenoside Rg1 from *Panax ginseng*. Japan J Pharmacol. (1990) 54:447–54. doi: 10.1254/jjp.54.447, PMID: 2087006

[43] Song Z Xie K Zhang Y Xie Q He X Zhang H. Effects of dietary ginsenoside rg 1 supplementation on growth performance, gut health, and serum immunity in broiler chickens. Front Nutr. (2021) 8:705279. doi: 10.3389/fnut.2021.705279, PMID: 34912836 PMC8667319

[44] Choi WJ Kim JH Han GP Kwon CH Kil DY. Effects of dietary hatchery by-products on growth performance, relative organ weight, plasma measurements, immune organ index, meat quality, and tibia characteristics of broiler chickens. Anim Biosci. (2021) 34:1181. doi: 10.5713/ab.20.0755, PMID: 33561330 PMC8255869

[45] Ma X Chi X Yuan L Wang Y Li Z Xu W. Immunomodulatory effect of ginseng stem-leaf saponins and selenium on Harderian gland in immunization of chickens to Newcastle disease vaccine. Vet Immunol Immunopathol. (2020) 225:110061. doi: 10.1016/j.vetimm.2020.110061, PMID: 32422443

